# Improving radiology reporting locally and globally: who, how, and why?

**DOI:** 10.1093/bjr/tqae253

**Published:** 2024-12-19

**Authors:** Kirsten L Gormly

**Affiliations:** Jones Radiology, Adelaide, SA 5063, Australia; Faculty of Health and Medical Science, The University of Adelaide, Adelaide, SA 5000, Australia

**Keywords:** template report, radiology referral, image quality, improvement, imaging guidelines

## Abstract

The radiology report is the communication from radiologist to referrer, used to inform prognosis and guide patient management. The report is the final step in a process which is influenced by the information on the referral, image quality, the reporting environment, and appropriate detection and interpretation of findings by the radiologist. It should present accurate, complete information in a way that can be easily understood. Even small improvements in any of these areas can have a significant impact on the average quality of radiology reports, with potential impact on vast numbers of patients across the globe. How do we train our future referrers to understand the complexities of imaging and write better referrals? How do we improve image quality as close to source as possible by engaging with equipment vendors? How can we make it easier for all radiologists to have access to the latest guidelines and use reporting templates where appropriate? Every radiologist has a role to play, with possible actions ranging from individual choice to departmental policies and global collaboration. The diseases we diagnose are the same, the equipment similar and knowledge freely available. All our patients deserve the best report we can provide.

## Main article

Imaging has become integral to the medical journey for many patients. From diagnosing a broken bone to staging cancer and assessing cerebral function, the images and report are used by doctors all over the world to guide treatment and inform prognosis. There are continuous advances in imaging technology and our understanding of what we see, but while the advances in our field are exciting and deserve attention, the majority of radiology reporting throughout the world every day is more mundane. There are frequent complaints about the quality of the images and reports submitted by “outside institutions” to a central multidisciplinary team (MDT) meeting or expert centre, with scans often performed and reported by people who are not subspecialized in that area. These issues occur within the most developed cities in the world, as well as some regional and underdeveloped locations.^1^^,^^2^ In many cases the equipment used is equivalent and the knowledge required to report is freely available via multiple online resources. Poor quality images and inaccurate reporting can significantly impact our patients, leading to inappropriate treatment choices and detrimental patient outcomes.

**Figure 1.**
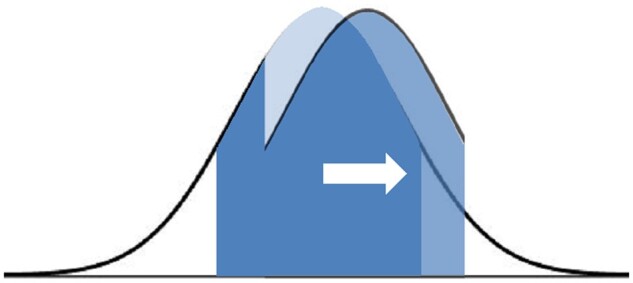
Considering the overall quality of images and reports by volume, the majority are in the central portion of the curve, rather than the far right where the latest developments occur at expert centres. By focussing improvement on the larger volume of everyday “average” reporting, even a small improvement leading to a shift to the right, can benefit significant numbers of patients.

Considering ways to make accurate, efficient reporting as easy as possible for all radiologists, we need to particularly consider this large component of routine, non-expert reporting, where even a small improvement could have significant impact across many thousands of examinations (Figure 1). Everyone has a role to play, whether you are a registrar, consultant, work in private or public practice, a section/department lead, or national leader, every interaction and initiative can produce an incremental benefit to patients. So how can we improve the quality of the radiology report both locally and globally?

If we consider the final clinical communication created through the report, there are multiple contributing elements including the referral, image quality, reporting environment, detection and interpretation of findings, and the report format. All of these provide opportunities for improvement.

## Referral

A detailed referral including information about the patient’s symptoms, provisional diagnosis, and past history, facilitates the optimal test, protocol, and interpretation of findings, yet clinical details are often minimal. Let us consider who writes these referrals and how we teach them. The majority of doctors will request imaging but have limited exposure to radiology during training.^3–5^ A study of Scottish Universities showed 0.3% of teaching hours were timetabled to radiology, with in hospital teaching ranging from 0 to 31 hours and noted “Radiology teaching ‥falls short of‥ GMC expectations of medical students at graduation”.^3^ An Australian study found that only 43% of junior doctors were aware of non-contrast, arterial, portal venous, and delayed phases on CT, and 49% felt somewhat or very unprepared to order CT investigations on starting clinical duties.^5^ Image interpretation is very important for doctors working in acute and remote environments, but doctors also need a deeper understanding of how imaging investigations are performed and interpreted within a clinical framework, and protocols are tailored to answer a specific clinical question. An inappropriate test or vascular phase has the potential to completely hide a significant abnormality.

A greater understanding of the imaging process may be gained by direct onsite exposure of medical students to CT, MRI, and ultrasound scanning. This could provide a practical and memorable experience, demonstrating the large number of CT and MRI protocols which can be selected by the technologist depending on the clinical question, the physical limitations of scans, and what patients experience. In combination with clinical teaching of appropriate investigations, this could instil the reason for writing good referrals and improve their ability to explain imaging tests to their patients, items included in the relatively new undergraduate radiology curriculum published by the Royal College of Radiologists.^6^ As little as half a day per modality could be effective, and while this requires time and effort from an imaging department, if the outcome is more complete referrals and better prepared patients, the potential time savings and improved patient care suggest this is a worthwhile investment.

In many countries, non-radiology specialist training beyond medical school includes an imaging component, but the content is designed and examined by non-radiologists. Valuable clinical experience gained during specialist training includes learning when to supplement good clinical judgement with diagnostic tests. However, with rapid imaging advances and increasing complexity, our colleagues are often unaware of new or varied imaging options, and benefit from our guidance on the most appropriate test and protocol for their clinical question. Imaging training by non-radiologists can perpetuate incorrect and outdated knowledge, as well as the attitude that they do not need radiologists to discuss imaging. This is exacerbated by the immediate availability of images before reports are released. While many specialists are very good at assessing images in their area, they often lack the nuances of interpretation or ability to detect other findings, which may have clinical and medicolegal implications.

As ward rounds no longer stop by the radiology department to discuss films, and many MDT meetings have stayed virtual following COVID, it is becoming more difficult to establish and maintain radiologist-clinician relationships. Radiologists are often “forgotten” when organizations are writing guidelines or designing clinical trials, leading to inappropriate recommendations and a lost opportunity to incorporate imaging advances. Creating formal bonds between radiology representative medical bodies and speciality organizations is needed to provide more meaningful and enduring change. Radiologist involvement in the content and teaching of imaging for non-radiology trainees would be a step towards increased understanding of the imaging options, referral requirements and the need for an ongoing partnership between treating clinicians and radiologists.

These efforts would better equip new generations of medical students and trainees for their future roles as imaging referrers and collaborators.

## Image quality

A radiologist can only report what is visible, and poor quality images can reduce accuracy of observations leading to a missed, incorrect, or incomplete diagnosis. MRI protocols are a good example where, despite published recommended optimal sequences and parameters, there remains great variability across magnets.^7–11^ All magnets have default protocols when they leave the factory, which are frequently altered by vendor application specialists, and in most expert centres are further altered significantly to achieve best practice, and then push the boundaries. These changes require knowledge, interest, and time by technologists and radiologists. It is also a wasted opportunity. There are a relatively small number of radiology equipment vendors globally, with their products used in a wide range of clinical settings all over the world. What if magnets had clinically appropriate protocols present at installation?

**Figure 2.**
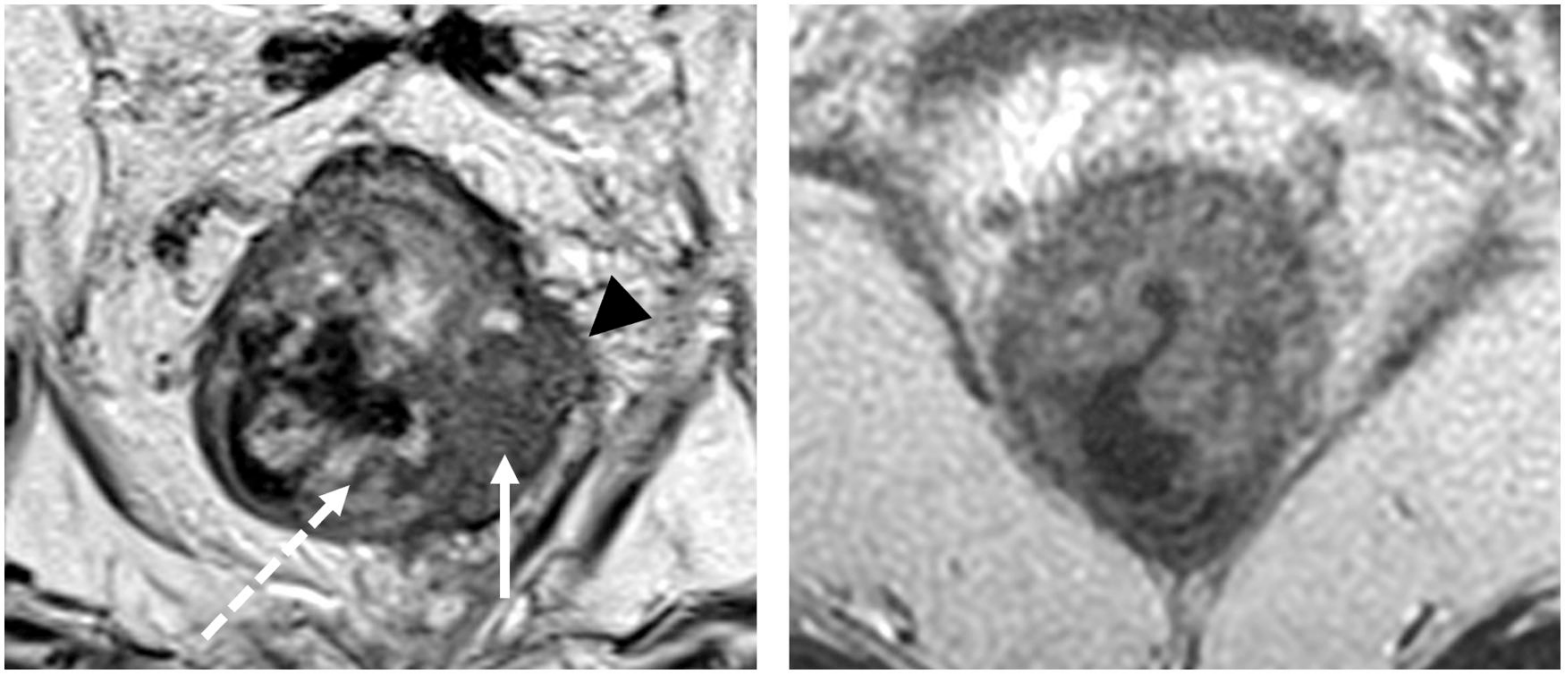
MRI rectum high resolution (HR) T2 sequences at 1.5 T demonstrates variation between an expert centre and peripheral site. High spatial resolution is required in rectal cancer to adequately differentiate the layers of the rectal wall and tumour versus normal structures. Many sites are unaware their scans are suboptimal. (A) HRT2 MRI at expert centre meets the scan parameters recommended in Australian and other national guidelines [slice thickness 3 mm, field of view 180 mm, matrix 320 × 320, voxel 1 mm^3^, 4 signal averages] with clear definition of the tumour (white arrow) normal submucosa (dotted arrow) and extension through the muscularis propria (arrowhead). (B) HRT2 MRI at peripheral hospital submitted as baseline for trial imaging, does not meet recommended criteria for the high resolution scan and is clinically suboptimal in differentiating the tumour and rectal wall [slice thickness 3.2 mm, field of view 220 mm, matrix 256 × 256, voxel 2.36 mm^3^, 1.5 signal averages.]

Many published best-practice protocols are achievable on working magnets. Some sequences will take longer on an older magnet to achieve a similar quality, and some may not reach it, but can come close. There are varying departmental requirements of speed versus quality in different settings, but ideally all would meet minimum standards. Non-experts are often unaware their images are below standard and simply report from what they have (Figure 2). Radiologist and technologist educators struggle with the difficult task of encouraging meeting attendees to change their inappropriate protocols when returning home. Improving the current factory release protocols at source, could significantly improve global image quality, which can lead to improved interpretation and reporting, with a flow on to patient care. High end research centres will continue to push the boundaries with individual variations, but it is in the non-expert centres where even small improvements on a large scale would have a significant impact.

Why does this not happen currently? On discussion with MRI development and education personnel at several vendors, it was discovered that many protocols have no direct clinical radiologist input prior to release. Few vendor applications specialists have formal clinical training around the protocols they are improving, and lack an understanding of which sequences are the most clinically important and what the radiologist needs to see [personal communication]. Direct involvement of expert radiologists and technologists in central protocol development and global applications specialist training would be welcomed. Vendor engagement and action in this space has the potential for widespread dissemination of significant image quality improvement.

## Reporting environment

Much has been written about reduced accuracy and efficiency associated with radiologist interruption and distraction. The intellectually demanding task of reviewing and analysing an ever-increasing number of images per study requires concentration. Proposed solutions at a local level include redesigning workflow and the reporting environment, which will not be further addressed in this article.^12–14^

## Detection and interpretation

As technology and research advances, the amount of knowledge is becoming too vast to be remembered by any single radiologist, reporting a range of body areas across their career. The younger generation are used to information being readily available at the touch of a button or voice command. Group knowledge, where experts analyse the evidence and provide recommendations on the best ways to detect and interpret lesions, include guidelines which are currently often continental, and the multiple -RADS (-reporting and data system) which have global representation and use. These consensus guidelines and systems are periodically updated to include new evidence.

A radiologist using such guidance during reporting is tapping into the wider knowledge of a community of experts, which should produce improved detection and analysis of their case. However, this information can be difficult to remember, may be remembered inaccurately, and updates can be published that the radiologist is unaware of. For optimal use, the current relevant information needs to be easily available when reporting. Having to open another website or resource to find guidance takes time, and introduces a distraction to the task of evaluating the images.

Reported barriers to using these tools include lack of availability, lack of familiarity, and lack of use by other specialists.^11^^,^^15^ On the latter point, who should be driving changes in reporting? If radiologists are aware of a system such as LI-RADS that increases our interpretation accuracy, should we avoid using it because the referrers are not familiar with it, or should we be actively promoting it as our duty to patients? Proactive use of the best tools we have available and encouraging the incorporation of these into clinical practice, seems appropriate in many cases. Radiologist-driven specialist imaging education, non-radiologist-clinician involvement in imaging guideline formation, and increased international representation may also improve uptake of new reporting systems. There are ongoing opportunities to consolidate continental guidelines that are currently duplicated, due to the similarities of imaging and knowledge globally, while still allowing for local variation in treatment strategies.

Reporting and dictation software vendors are beginning to recognize the value of incorporating consensus guideline recommendations and staging information into their systems. This may be activated by mention of a particular finding in the report, a click or voice command, providing the reporting radiologist with instant access to the current version of classifications, staging, and follow up recommendations. Given the relatively small number of continental and global guidelines and potential for further consolidation, it should be possible to have these incorporated into systems and select your local preference. It is hoped this becomes an integral part of these systems rather than an additional purchase, with updates proactively identified by vendors and pushed out to users, similar to software updates in a wide range of computer operating systems and applications.

A burgeoning number of computer-assisted and artificial intelligence tools can also improve detection and interpretation, which are beyond the scope of this discussion. Ensuring these products match clinical need and do not have unintended adverse consequences requires radiologist involvement during development and implementation.

## Report format

The radiology report is the method of communicating the imaging findings to the treating doctor, and is the culmination of all prior efforts to produce a correctly protocolled, diagnostic quality scan, with detection and appropriate interpretation of the findings. Treating clinicians receiving all the information they need in a manner they can easily understand is vital for optimal patient care. Radiologist training and experience are important factors in producing a high quality, clinically relevant report. It is well documented that using a template report has benefits including increased completeness of necessary information, a concise and consistent format and improved referrer communication, as well as increased ability to data mine for research. They are particularly useful in oncology imaging, and known diagnoses with required information, but may not be appropriate in all settings. A poorly designed generic template that does not address the clinical question can be equally detrimental. Many international societies recommend the use of structured reports, but studies have shown variable uptake by radiologists, with barriers including experience, training, a fear of loss of autonomy, and availability of templates.^11^^,^^16–20^ There are numerous freely published national and society template reports^21–24^ but these are not provided with many reporting software platforms. For an institution or individual to have a template in their standard voice recognition (VR) system it needs to be entered, either individually by a radiologist or centrally in a department, using valuable time and resources. The fact that multiple individuals and centres around the world are currently replicating this process to make a template available is a waste of resources and lost opportunity.

The ESR recently highlighted the need for practical solutions to be provided by vendors.^16^ The incorporation of national and global published templates into systems at source, prior to installation or upgrade at sites, would make template reporting far easier to promote to radiologists and save resources within departments. This would still allow individuals and institutions to install their own, or tailor the template according to local preferences, but provides an accepted starting point. To facilitate vendor provision, radiological groups could collaborate further to reduce the number of recommended base templates, as disease processes have a generally agreed set of information required for clinical decision making, and many current templates have minimal variations.

Provision of templates with software will improve availability, but the individual radiologist still needs to choose to use it, highlighting the importance of individual actions alongside systematic change. Overcoming the barriers of lack of experience, training, and availability, can be addressed at local departmental level with provision of training, encouragement, and possibly a departmental directive regarding template use. Optimal reports also benefit from an understanding of local practice where this is feasible. Ongoing collaboration with clinical teams will guide reporting the required information in the most effective way. The ESR paper proposed consideration of wider directives and a potential need for financial incentives to promote template reporting.^16^ In Australia, pathology structured reports have been successfully mandated at a national level since 2022.^25^^,^^26^

## Why is improvement so slow?

Many people discussing the issues presented above comment that it is all too difficult. They state you will never get experts to agree, vendors to assist, or individuals to change. The issue of expert agreement misses the point. So many centres in the world are not scanning or reporting in an expert setting, their images are of reduced quality and the radiologists either cannot remember, or do not know all the details needed for a high quality report. Expert international panel agreements have been reached in the -RADS, providing genuine assistance to radiologists around the world. We are not aiming for complete consensus at the top, but enough agreement to provide guidance on minimum standard protocols and reports, which can then be distributed for easy access via vendors and software updates.

People assume vendors are resistant to these ideas, but they have shown a genuine willingness to engage. While it seems slightly unbelievable there has not been more clinical input to date, any advance requires initiation and momentum.

Individuals can change, particularly if they are supported to do so and change is made easy and efficient. While financial incentives and links to reimbursement are more likely to succeed, if a reasonable majority of individual radiologists choose to change, we could be spared such draconian measures. The easy availability of staging information, classification systems, and a template identifying the areas required for a clinically adequate report may seem aspirational, but is entirely achievable using current technology.

Just because something is difficult does not mean it should not be attempted. Most people genuinely want to do their best, they just do not have the time, tools, or knowledge to do so. Every one of us can make a small step to contribute to the whole.

## Driving the change—who, how, and why?

Even a small improvement in each of the areas discussed above should result in improved quality of the “average” radiology report created daily in non-expert centres around the world (Table 1). Global improvement is possible as we all report the same diseases which often appear in expected patterns, use similar machines produced by a small number of international vendors, with increasing use of global VR and reporting software. By focussing on the “average” report, which forms the majority of practice around the world, the potential scale of uplift is greater.

**Table 1.** Areas contributing to the radiology report, issues, and opportunities for improvement at local and global levels.

	Issue	Opportunity	How
Referral	Incomplete clinical information Lack of understanding by referrers of the imaging process	Increased training and exposure of medical students and non-radiology registrars to the medical imaging process	Student placements with technologists observing patient flow and scans Formal radiology input into imaging content of non-radiology registrar training
Image quality	Poor quality images at non expert centres Radiologists unaware of reduced quality Limited time or knowledge to improve locally Poor quality images influence report accuracy	Have clinically recommended protocols installed at factory release Improve knowledge of global application specialists for wider dissemination Increased feedback of image quality issues locally	Radiologist and technologist input into protocol design with vendors Clinical education of application specialists Local encouragement of radiologist feedback on image quality Local departmental support for optimization
Reporting environment	Interruption and distraction affect concentration, report accuracy, and efficiency	Provide appropriate physical and mental environments for reporting	Local optimization of departmental workflows and reporting spaces
Detection & interpretation	Individuals unable to remember all required knowledge and be aware of updates Radiologists do not use guidelines proven to optimize assessment Detection misses on the large numbers of images and series	Have relevant information seamlessly available at reporting with automatic updates AI and computer assisted detection products targeting common and important misses	Promotion to vendors of need to incorporate latest guidelines into reporting platforms Radiologist input into detection software development to match clinical need Local availability, and encouragement to use guidelines Individual radiologist choice
Report format	Template reports increase accuracy and completeness, but are infrequently used Installing templates is time intensive and often done at site Radiologists are resistant to using templates	Provision of templates with reporting software Consolidate national and continental templates Increase availability, training, and expectation of use	Promotion to vendors to incorporate templates at source Local availability and training in template use Consider local and wider directives for template use Individual radiologist choice

**Figure 3.**
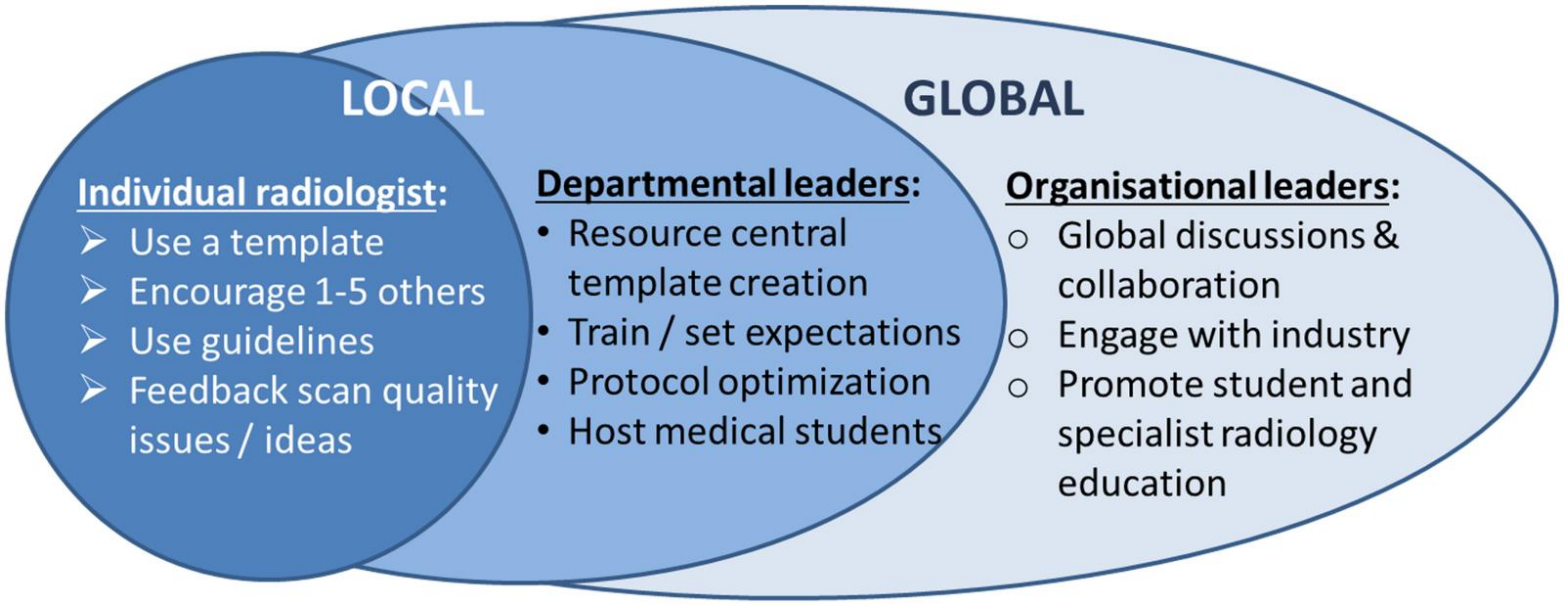
Everyone can play a role in improving radiology reporting locally and globally.

We can all take some action towards improvement (Figure 3).

- Individual radiologists can choose to follow international guidelines, report with a template where appropriate, highlight poor image quality, and encourage their colleagues to do the same. They can also contribute to teaching medical students and specialist registrars.
- Departmental directors and others with local influence can resource central creation of templates, provide training, and consider directives regarding their use, invest time in scan protocol optimization, and host medical students in the departments to expose the next generation of referrers to the realities of imaging.
- Those in leadership positions in national and international organizations can encourage global collaboration in guideline and template formation, engage with industry to provide clinical radiology input into product development and education, encourage key information and template provision in reporting software with regular updates, and approach other specialist organizations to formalize radiologist involvement in the teaching of imaging.

Why? Because all the patients we image deserve the best report we can provide.

By improving referrer training, using the information and best practices that already exist, promoting systematic improvements as close to source as possible and taking individual action, we can make the “average” report better for all our patients, both locally and globally.
